# After Hospital: Should Older Care-Needing Patients Be Transferred to Their Homes or to an Intermediate Care Institution?

**DOI:** 10.3390/healthcare10030475

**Published:** 2022-03-03

**Authors:** Heidi Gautun, Linda Aimée Hartford Kvæl, Christopher Bratt

**Affiliations:** 1 Norwegian Social Research, NOVA, Oslo Metropolitan University, 0130 Oslo, Norway; linaim@oslomet.no; 2 Department of Psychology, Inland Norway University of Applied Sciences, 2624 Lillehammer, Norway; christopher.bratt@inn.no; 3 School of Psychology, University of Kent, Canterbury CT2 7NZ, UK

**Keywords:** collaboration, acute hospitals, community health and social care services, intermediate care, older patients, continuity of care

## Abstract

Background: In most European countries, communities need to provide health and social care services to an increasing number of severely ill patients discharged from hospitals. We investigated whether nurses in hospitals and in the communities’ health and social care services experienced that the administration in the municipalities allocated older patients the right type of services after hospital discharge. Methods: We used data from Norway, with a qualitative pilot study and quantitative analysis (structural equation modeling) of surveys involving 2431 nurses on inpatient wards in acute hospitals and 4312 nurses working in nursing homes or home nursing. Results: Dissatisfaction was widespread with the use of patients’ homes the first days after hospital discharge. Among nurses working in hospitals, 38% were commonly or very commonly disagreeing with the use of the patient’s home after hospital discharge, 25% among home nurses, and 18% among nurses in nursing homes. Home nurses were more prone to oppose the use of patients’ homes if they also experienced that their service had inadequate staffing or inadequate medical equipment. Conclusions: This research indicates conflicting priorities between the bureaucracy and nurses involved in actual work with older patients. From the nurses’ perspective, the municipalities’ administration was offering too few older patients short-term-stay in an intermediate care institution as part of the clinical pathway from hospital to home. However, providing more recourses to home nursing would improve their ability to provide sufficient care to older patients discharged from hospital.

## 1. Introduction

Older patients often need continued care after they have been discharged from hospital. Where should these patients be transferred to: directly to their homes or first to an intermediate care institution? Allowing older patients in need of care to stay at their homes has personal and financial advantages [1]. Yet some patients may be too ill, or the services involved in home nursing may not be well equipped: they may lack adequately trained staff, or they may lack medical equipment to provide care and treatment for older patients discharged from hospitals.

The current research focuses on experiences from Norway. In Norway, an “allocation office” in the municipality administration uses information they receive from the hospital along with preferences expressed by the patient to decide which service the individual patient shall receive when they are discharged from hospital. Notably, the healthcare professionals in the hospital have no say on what the decision will be, apart from providing information on the patient. This paper investigates whether nurses in hospitals and in the communities’ health and social care services agree or disagree with decisions made by the allocation office.

Data were collected among nurses who were involved with older patients discharged from hospitals and then transferred to community health and social care services; we include both hospital nurses and community nurses in our data. A qualitative pilot study uses interviews with hospital nurses. The main study uses surveys with more than 6500 nurses, comparing views among three groups of nurses (nurses at hospitals, in home nursing, and in nursing homes), and we investigate potential predictors of why nurses in home nursing may oppose that their service receives older patients discharged from hospitals. 

### 1.1. Bridging the Gap from Hospital to Going Home

Most patients who receive health and care services both at hospitals and in the communities are patients 80 years or older. This is true for our example of Norway and for the OECD countries in general [2]. Some of these patients will have complex care needs and be particularly vulnerable when they depend both on hospital treatment and subsequent follow-up care by the communities’ health and care services. Due to their complex needs, their care can easily become suboptimal [3,4,5]. Many of these patients need intermediate care in an institution after hospital discharge before they return home [6]. Allowing older patients to stay *temporarily* in an institution providing intermediate care might therefore be a crucial contribution to the policy of “aging in place”, allowing people to live as many years as possible in their own homes [7].

Norwegian health services sometimes use this strategy, providing intermediate care to bridge the gap between hospital treatment and home care among older care-needing patients. The patients can, for a short period, receive intermediate care in an institution such as a nursing home, based on an overall aim to enable older people to live at home as long as possible [7]. The question raised in the present research is whether too few receive such intermediate care.

This issue has received limited attention: different health facilities may disagree about which services older people should receive when discharged from hospital. Earlier research has highlighted that communities’ health and social care services experience that patients are discharged prematurely from hospitals [8,9]. Yet some evidence also suggests that hospital nurses may have concerns when older patients are transferred to their homes rather than to an institution for intermediate care: in qualitative interviews from 2019, 14 Norwegian hospital nurses indicated that too few patients received intermediate care in an institution, such as a nursing home [10]. How widespread are such concerns; do the few interviewed nurses reflect a widespread concern among hospital nurses? Equally, little is known about what nurses in the community services think of decisions to send discharged older patients home directly after hospital treatment.

### 1.2. Declining Length of Hospital Stays

The length of hospital stays has been steadily reduced in most Western countries, from the 1960s and onwards [2,11]. This is particularly true for Norway, where hospital stays are now shorter than in most other European countries and much shorter than the average for all OECD countries: the average stay at hospitals in 2017 was 6.5 days in Norway and 8.0 days in all OECD countries [2]. Two reasons for steadily shortened hospital stays are improved treatment methods and advances in medical technology [12]. These improvements have resulted in hospitals treating a growing number of inpatients within a shorter period and simultaneously also an increasing number of outpatients.

The declining length of hospital stays is also driven by a policy of decentralizing care and treatment. This trend seen in many countries has drawn ideas from new public management [13]. In Norway, it resulted in various reforms and regulations in the 1990s, moving many health and social care services to the communities [8,14,15]. Specifically, ideas from new public management inspired a *purchaser-provider model* in Norwegian health and social care services [16,17]. To ensure that those who allocate services do not also provide these services themselves (which might lead to self-serving decisions), several Norwegian municipalities have introduced a model that separates the role of ordering services from providing the services. An allocation office in the municipal administration, i.e., the purchaser, assesses and approves services for each individual patient. The providers, i.e., healthcare professionals working face to face with the patients, have minimal influence on the decisions made by the allocation office. One consequence of the purchaser-provider model is that it requires standardization of pre-defined tasks, imposing a more rigid time regime, easily resulting in less emphasis on individualized care due to the lack of professional discretion among the “providers” [18].

Applying concepts from private sector management was meant to make decentralized health and social care services financially more efficient. Yet the decentralized health and social care services were also presented as in the patients’ best interest: moving care to lower levels ensures that more patients receive care and treatment within their homes. To be successful, however, such a decentralization requires that community health and social care services can provide advanced care [14].

The last point is crucial. Specifically, can advanced health and social care services be delivered in people’s homes? The policy of “aging in place” and the de-institutionalization of care have led to increased demands on home care services [19,20]. In Norway, 2020, one-third (28.9%) of the population over 80 received home care services [21]. Simultaneously, 11.4% of the population over 80 years were residents in a nursing home [22].

### 1.3. Patients Ready to Be Discharged Remain in Hospitals

Earlier research in Norway has indicated that staff in hospitals and in the municipalities easily disagree on when patients have completed necessary hospital treatment and are ready to be transferred to the community services [3]. The hospitals decide when patients are ready to be discharged, while the municipality decides the kind and the scope of service offered to patients after hospital discharge. One study found that within the community health and social care services, approximately half of the nurses reported that more than 20% of the patients were discharged prematurely from hospitals [23].

In many municipalities, health and social care services may not have the capacity to provide care for all patients in a timely manner after hospitals have declared patients ready to be discharged. Norway has introduced legal and financial incentives to prevent patients from remaining in hospitals [24], but since municipalities do not always have the means to provide sufficient health care after patients are discharged from hospitals, some patients remain at hospitals, and the municipalities pay considerable fines [25].

### 1.4. Aim of This Research

The present research has two aims: (1) investigate whether nurses in hospitals and in the communities’ health and social care services experienced that the administration in the municipalities allocated older patients the right type of services after hospital discharge; (2) investigate predictors of home nurses’ disagreement with the decision to transfer patients directly to their homes.

## 2. The Current Research

This research uses two studies; a qualitative pilot study and a quantitative study based on surveys. The qualitative pilot study uses interviews that allowed nurses in hospitals to speak freely on their experiences when older patients were discharged. The main study uses quantitative analyses of data from surveys among over 6000 nurses in hospital and the municipalities’ health and social care services.

### 2.1. Qualitative Interviews

We conducted qualitative interviews with four hospital nurses separately. The nurses worked at three different hospitals. All had several years of experience in hospitals, working either in a medical, surgical, or geriatric department. Two of the nurses were selected by The Norwegian Nursing Organisation (NNO), two by the head of a research unit at one of the hospitals. In both cases, the selection of nurses was based on our request to meet informants who worked at inpatient wards with older patients and had experienced discharge of older patients to the municipalities’ health and social care services. The three hospitals had different profiles in terms of size and ownership and are located in or outside the capital Oslo.

### 2.2. Quantitative Surveys

The quantitative surveys build on earlier research as well as findings in the qualitative pilot study. We seek to assess how common (or uncommon) it is among each group of nurses to disagree with the allocation office’s decision to send a specific discharged older patient (65 years or older) to their homes with home nursing. Conversely, we assess how common it is to disagree with sending discharged older patients to an institution for intermediate care. We compare responses among three groups of nurses: nurses at hospitals, in home nursing, and in nursing homes.

Finally, we develop an analysis of variables predicting opposition among home nurses to the use of home care for older patients discharged from hospital. Since the decentralization of care imposes demands for advanced care within homes, we expect that home nurses’ opposition to sending discharged older patients to their homes is linked to inadequate resources in home nursing: both inadequate staffing and inadequate medical equipment. We also test other potential predictors, such as whether the nurses hold a leadership position, have further education, and their work experience in years.

## 3. Qualitative Pilot Study

All interviewed hospital nurses indicated that they occasionally disagreed with the decision made by the municipality’s allocation office concerning where an older patient should go after discharge. Specifically, the nurses indicated opposition to the decision to deny very ill older patients a stay in an intermediate care institution and that several such patients had to be readmitted to the hospital shortly after discharge. Sometimes a hospital decides to reverse a decision to discharge a patient, thereby avoiding that the patient was sent to the allocation office’s choice of service. Two of the interviewed nurses (working at different hospitals) added that standing up for the patients’ needs could easily result in quarrels with the allocation office. One strategy became to avoid communicating the genuine needs of the patient to avoid conflicts with the allocation office.

According to the interviewed nurses, many of the executives at the allocation offices did not have the necessary medical competence to evaluate patients: some were lawyers, economists, or they had another professional background not related to nursing. So, the allocation office could fail to realize how serious a patients’ medical condition was. One example was an older patient with a hip fracture who had been allocated home care services after discharge. The nurse explained:

“Not all executive officers understand what a hip fracture is. This patient lived alone and was left in his bed in his apartment. In the evening they put a diaper on him, then they came back in the morning to change the diaper. In an intermediate care institution, that patient could have been helped to the toilet day and night. I asked the allocation office: ‘Do you want me to tell the patient to pee on himself?’, and she answered Yes! This has happened frequently with other patients as well.”

## 4. Surveys: Materials and Methods

### 4.1. Samples

Building on the qualitative interviews, we conducted two nationwide, web-based surveys in Norway, both in 2017: one among hospital nurses working on inpatient wards in somatic hospitals (*n* = 2328 in the final sample analyzed), one among community nurses working either in home nursing (*n* = 2112) or in nursing homes (*n* = 2169). Participants included only nurses who had been involved in the transferral of patients 65 years or older from hospitals to the community health and social care services. We excluded nurses who worked in administrative positions, in other services, or did not treat older patients. We also excluded nurses working in emergency rooms or outpatient clinics.

We aimed at having a large, nationwide sample rather than a small sample from a few services with uncertain implications for other municipalities. Since no national register is available to identify nurses fitting our selection criteria, we employed e-mail lists of nurses who were members of the Norwegian Nurses Organisation (NNO), where most Norwegian nurses are organized. We sent emails to all members of the NNO registered as working in acute hospitals (29,316 nurses) and members registered as working in the municipalities (20,714 nurses). Apart from highlighting our target group of nurses and a link to the online questionnaire, the emails included a recommendation by the NNO to participate in the survey. To ensure that only nurses fitting the inclusion criteria were included in the analyses, the questionnaire asked the nurses about their current workplace and whether they had been involved in the transition of older patients from hospital to community services.

Given the procedure used to ensure large and nationwide samples, many nurses received the e-mail without being part of the target group. The procedure used also prevented us from calculating response rates. The lists of NNO members do not contain information identifying how many of the nurses met the inclusion criteria, and the member registers in the NNO are not updated regularly. Consequently, some of the emails will have gone to nurses no longer belonging to the target group, such as nurses who had changed workplace, returned to education, stopped working in the services, were on leave or long-term sick leave, or were now receiving disability pension, or were retired. In addition, the many nurses who had not been involved in the transferral of older patients from hospitals to nursing homes or home care were asked to disregard the questionnaire. The inability to compute response rates was more than compensated for by the current design’s ability to include a very large sample of nurses (total *N* = 6609), resulting in data being more representative than other data collection methods would: participating nurses represented 88% of Norway’s 428 municipalities; hospital nurses worked at different types of acute inpatient wards, and at wards of different sizes; and community nurses worked in municipalities of various sizes, from large cities to municipalities with less than 500 inhabitants. Half of the community nurses (49%) were employed in nursing homes, 45% worked in home care services. The remaining nurses were employed both in nursing homes and home nursing; we dropped these nurses from analyses. Descriptive statistics for the samples are available in Tables S1–S4, included in the Supplementary Materials (link is available towards the end of this article).

Along with the qualitative interviews, both surveys were approved by the Norwegian Centre for Research Data, project numbers 52,722 and 53,155. Participating nurses were guaranteed anonymity, and answering the questionnaire was considered as informed consent. We sent three reminders: one, two, and three weeks after the original invitation to participate in the survey.

### 4.2. Measurements and Analysis

The questionnaire items built on findings in the qualitative pilot study and on earlier research on discharging older patients from hospitals [8,26,27]. To further validate the questionnaire items, we first tested them on random samples of 20 nurses in hospitals and 41 nurses in the community services before emailing the questionnaires to nurses in the NNO.

The surveys assessed *how common it was for the nurses to disagree with the decision* to transfer discharged older patients to home nursing and institutions, respectively. These items were introduced with the following sentence “When older patients are discharged from hospital—how common or uncommon is it that”, followed by one item stating “you disagree with the allocation office’s decision to offer home care services instead of a place in an institution”, the other stating “you disagree with the allocation office’s decision to place older patients in an institution instead of providing home care services”. Both items used a five-point response scale with the following possible responses: Very uncommon; Uncommon; Sometimes; Common; Very common.

We also assessed *background variables*: age, sex, years of experience as a nurse, years working at the current workplace, and working hours. Details on these measurements are available in the Supplementary Material, Section 1, Tables S1–S4. We further included an item on whether the participating nurse held a *leadership position* (dichotomous variable; no or yes) and whether the nurses had received *further education*, with four alternative answers: No; One year or less; More than one year (but not master); Master. In addition, a “Don’t know” option was included, coded as missing data.

In the survey sent to community nurses, we also included four items on the current *staffing of their own service*. These items were introduced with the following sentence: “To what extent do you agree or disagree with the following statements about how well equipped your service is to receive older patients who are discharged from hospital”. The four items were: “The number of unskilled workers is too high”; “The service is adequately staffed with nurses”; “Services are sufficiently staffed with other qualified care workers (e.g., licensed practical nurse and others)”; “There are too many vacancies”. All four items used a five-point Likert response scale (Completely agree; Partly agree; Neither agree nor disagree; Partly disagree; Completely disagree), as well as a “Not sure” option, which was coded as missing data. Views on the *available medical equipment* in the nurses’ own service were assessed with one item: “The service has the necessary medical-technical equipment”, again with a five-point Likert response scale.

**Figure 1:**
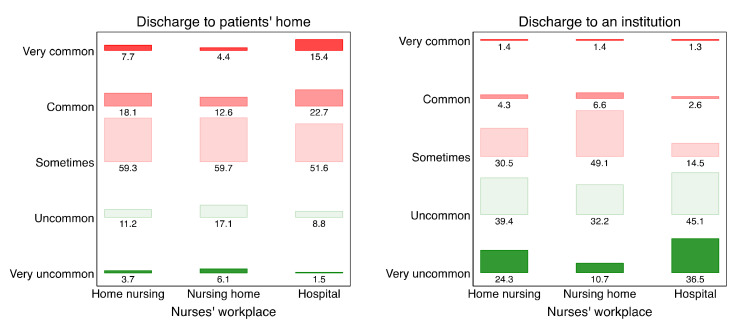
Disagreeing with transferring older patients to home nursing or to an intermediate care institution after hospital discharge.

Statistical analyses were performed with R version 4.1.0 [28] and the “lavaan” package in R [29]. Other R packages central to this research were “knitr” [30], “kableExtra” [31], and “semPlot” [32]. Figure 1 was developed with Stata 17, using the addon package “barplot” [33].

Analyses reflected that the dependent variables (disagreeing with the transferral of patients to their homes or to an institution, respectively) used five-point ordinal scales. In lavaan, analyses of ordinal dependent variables use probit regression with diagonally weighted least squares, DWLS. As demonstrated in the Supplementary Material (Section 2), item-level predictors with all four indicators of inadequate staffing would bias the results. Instead, we used structural equation modeling, SEM [34], where we estimated a latent variable of staffing problems with the four items as indicators.

SEM models were evaluated with fit indices, using commonly recommended cut-off values for indices of approximate fit [35]; specifically, the root mean square error of approximation (RMSEA, with values not above 0.06) along with its 90% confidence interval (the upper limit should not go beyond 0.08), and the comparative fit index (CFI, with a cut-off value at 0.95). Consistent with common practice, we did not emphasize the chi-square, given the large sample sizes. All analyses can be reproduced with the code included in the Supplementary Material (Section 4). The data are available online.

## 5. Survey Results

### 5.1. Views among Hospital Nurses

Hospital nurses indicated that they often preferred institutional care over sending older discharged patients to their homes where they would depend on home nursing (see Figure 1). Specifically, nine of ten hospital nurses (90%) indicated that they sometimes or commonly disagreed with the choice of patients’ home over institution. Even if one restricts the analysis to those expressing stronger disagreement, opposition against the use of patients’ homes was substantial: 38% of the hospital nurses responded that it was “common” or “very common” for them to disagree with the choice to transfer discharged older patients to their homes.

Opposition to using older patients’ homes was more frequent among hospital nurses than among either of the two groups of community nurses. These differences are illustrated in Figure 1; Table S5 in the Supplementary Material quantifies these differences with ordered probit regression: the strongest opposition was identified among hospital nurses as the reference group, somewhat less opposition was identified among home nurses, *b* = −0.33 (95% CI = −0.39, −0.26), and the least opposition was identified among nurses in nursing homes, *b* = −0.62 (−0.68, −0.55). Consistently, the hospital nurses rarely disagreed with discharging older patients to an institution, and they did so less often than either of the two groups of community nurses. (The ordered probit regression gave *b* = 0.42 (0.36, 0.48) for home nurses and *b* = 0.87 (0.81, 0.94) for nurses in nursing homes, again with hospital nurses as the reference group.).

### 5.2. Views among Community Nurses

Community nurses too disagreed more frequently with the use of home nursing over an institution. *Home nurses* more often than *nurses in nursing homes* disagreed with the use of home nursing (see Figure 1). In addition, the use of institutions for discharged older patients received more opposition among *nurses in nursing homes* than in either of the two other groups of nurses. Yet overall, opposition to the use of institution was rare.

Given the widespread opposition to the use of home nursing, we were particularly interested in explaining home nurses’ tendency to oppose the use of patients’ homes. We tested various predictors; details are available in the Supplementary Material, Section 3.1). The four items on staffing were used to develop a latent variable of inadequate staffing. Other variables having at least a minor contribution to explaining opposition to the use of patients’ homes included leader position, having further education, and whether the service had adequate medical equipment (according to the nurses’ views); see the Supplementary Material for details. However, the most relevant predictors turned out to be staffing, medical equipment, and whether the nurses had a leadership position or not.

**Figure 2:**
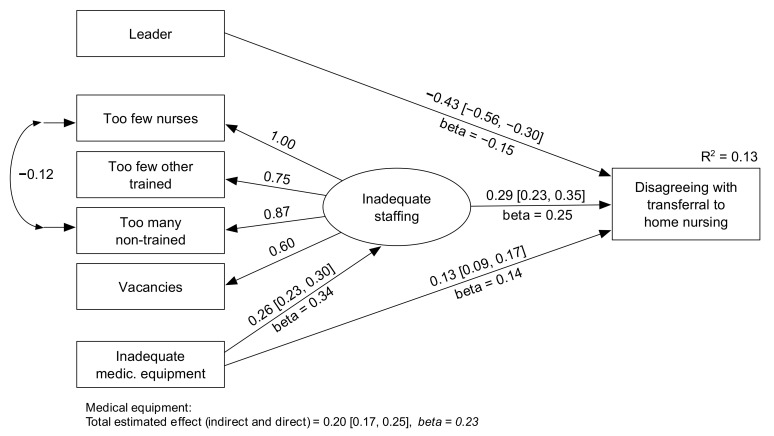
Home nurses disagreeing with transferrals to home nursing. Fully standardized estimates are labeled beta: the remaining numbers are unstandardized estimates (some with 95% confidence interval). Estimated total effect for the variable “Inadequate medical equipment” was b = 0.21 (95% CI = 0.17, 0.25), beta = 23. All parameters had *p* < 0.001. The model had sufficient fit: scaled chi-square (11) = 63.653, *p* < 0.001, scaled comparative fit index = 0.982, scaled root mean square error or approximation = 0.049, with a 90% confidence interval from 0.038 to 0.061.

The outcome of this analysis is shown in Figure 2. The model fit the data well if it included a regression path from “inadequate medical equipment” to “inadequate staffing”. We added two robustness checks, both described in the Supplementary Material, Section 3.2. One tested a less reasonable model with suitable fit; the implications of the two models were similar. A second robustness check tested for a possible biasing method effect: a tendency to express disagreement/dissatisfaction. Adding an estimated method effect to the model confirmed that the results were not biased by a tendency to express disagreement and dissatisfaction.

The results shown in Figure 2 indicated that leaders in home nursing were moderately less likely to disagree with the use of patients’ homes. Staffing problems (as reported by the nurses) proved to be a substantial predictor (*beta* = 0.25). Equally, inadequate medical equipment was a substantial predictor of disagreeing with the use of patients’ homes: the total estimated effect of inadequate medical equipment was *beta* = 0.23 (with indirect effect via staffing problems added to the direct effect).

## 6. Discussion

The initial qualitative pilot study among hospital nurses revealed concerns that too few older patients received adequate follow-up services after a hospital stay. The subsequent quantitative analysis substantiated this finding with large, nationwide surveys. Hospital nurses, and also community nurses, frequently disagreed with the allocation offices’ choice to send older patients discharged from hospital to their homes rather than to intermediate care at an institution.

The analysis then focused on home nurses. Home nurses were more negative toward sending older patients to their homes if the home nurses experienced that their service had inadequate staffing or inadequate medical equipment.

### 6.1. The Need for Intermediate Care at an Institution

The current findings corroborate the suggestion that many older patients are likely to benefit from being moved to an intermediate care unit after hospital discharge rather than directly to home care services [9]. Previous research indicates that patients in need of community care and stay in hospital often are frail patients over 80 years with complex health issues [2,3,4,5,6]. The present research clearly suggests a re-evaluation of services provided for older patients after hospital stay. Even when the intention is that discharged older patients shall be able to live in their homes as long as possible, transferring them directly to their home is not always advisable: for frail patients, sufficient recovery time at an intermediate care institution can improve functioning and thereby prevent readmissions and a need for prolonged hospital treatment. Allowing older frail patients an intermediate stay at a care institution may also be more cost-efficient and reduce the overall need for health and care services for older patients [36]. Consequently, intermediate care service is a crucial part of providing patients with a suitable pathway [6,37,38].

Well-functioning collaboration between care levels when transferring patients from hospitals to their homes [39] is a national priority in many countries [40]. Yet guidelines for how to achieve this aim are still missing [36]. We suggest that providing intermediate care to older patients after hospital discharge may facilitate “aging in place” [41].

### 6.2. Conflicts between Nurses and the Bureaucracy

Findings from both the qualitative interviews and the surveys highlight a potential conflict between those who work in the services and staff in the municipalities’ allocation office. When earlier research or health authorities have referred to disagreements or conflicts between municipalities’ administration on the one hand and health personnel at hospitals or communities, on the other hand, they have rarely described how these conflicts evolved. The current research has identified a crucial source of such conflicts: substantial disagreement around *where* older patients should be transferred after hospital discharge.

When hospitals define a patient ready for discharge, the allocation office in the municipality chooses a follow-up health service for the patient. The idea is to choose the lowest effective level of care, which often results in allocating home services [42]. Yet nurses working daily with patients in hospitals or municipal services are probably more concerned about what they experience as the patient’s true needs. We interpret the tension between nurses and the municipalities’ administration as an effect of conflicting priorities among agencies within the purchaser-provider model [43]. Our findings thus support the growing body of literature arguing that the application of ideas from new public management should be reconsidered. Specifically, the purchaser-provider model applied to health and social care services may be less suitable than originally anticipated. This conclusion is in line, for instance, with an evaluation in the U.K. of three decades with new public management, where the authors argued that the outcome was an expensive and bureaucratic administration at the cost of overall resources available for the services [44].

### 6.3. Resources Available in Home Care and the Use of Patients’ Homes

The current research revealed that home nurses’ experiences of inadequate staffing and inadequate medical equipment predicted their opposition to sending discharged older patients to their homes instead of to an intermediate care institution. This is likely to be a widespread problem; resources available to the municipal health and social care services have not been increased consistent with the extended needs among patients after hospital stays have been steadily shortened [8,45,46]. Many home nurses were critical of the current practice, indicating that too few older patients received intermediate care at an institution after a hospital stay.

There is no reason to suggest that home nurses’ opposition to the use of patients’ homes might be driven by an attempt to limit their own work burden. Hospital nurses more often than home nurses opposed the use of patients’ homes, and even nurses in nursing homes expressed more dissatisfaction with the use of patients’ homes than with an institution. Using structural equation modeling, we also established that the current findings were not biased by a method effect stemming from some nurses tending to express negative views in general.

The present research, focusing on experiences among nurses, corroborates earlier assessments of experiences among patients, their families, and health care personnel. Such earlier research has referred to intermediate care as an important part of the clinical pathway [6,37,38,41]. If the municipalities are able to properly accommodate the needs of older patients discharged from hospital, then a suitable number of intermediate care places need to be made available in nursing homes, and the home care services must be equipped with improved resources.

### 6.4. Future Research

Strengths of the current research include the use of three different data sources (qualitative interviews with hospital nurses, a survey among hospital nurses, a survey among community nurses) and large samples in the two surveys. We also included a validity check by testing for a method effect (a tendency to express disagreement and dissatisfaction) and found no evidence of such a method effect.

However, the present research did not include assessments of patients’ perspectives, and it did not assess views in the municipalities’ allocation office. Both can be considered in future research. Future research may, for instance, compare views among staff at the allocation offices and their experiences with the transition of older patients after hospital discharge with experiences among community nurses. Another interesting line of research would be to apply a cost-benefit analysis to evaluate the effectiveness of intermediate care compared to home nursing for frail patients in the first days after hospital discharge.

## 7. Conclusions

The present research has highlighted that conflicts between the municipalities’ administration and nurses treating and caring for patients cannot be reduced to a problem of collaboration between care levels, as sometimes suggested. Instead, tensions between levels of service uncovered in this research seem to originate in conflicting priorities among practicing nurses and the bureaucracy. According to the nurses included in this research, the administration in the municipalities often fails to offer older patients the treatment they need. Too few are offered a stay in an intermediate care institution in the days immediately after their discharge from hospital. Even though the present research suggests that more older patients should be discharged to an intermediate care institution rather than to their homes, the results also emphasize that some patients might have their needs covered outside an institution if home care services have sufficient staff with a professional healthcare background.

## Data Availability

Code used in data management and analyses are available in the Supplementary Materials.

## Supplementary Materials

The following supporting information (which includes tables, figures, and code) is available in a single PDF file and can be downloaded at: https://www.mdpi.com/article/10.3390/healthcare10030475/s1, Figure S1: SEM model of home nurses disagreeing with the use of patients’ home, standardised estimates. Figure S2: SEM model with revised factor model, standardised estimates. Figure S3: SEM model of nurses in nursing homes disagreeing with the use of institution. Figure S4: SEM model with revised factor model, unstandardised estimates. Figure S5: SEM model with added method factor (tendency to disagree), unstandardised estimates. Table S1: Age of participants. Table S2: Years experience as a nurse. Table S3: Years at the current work place. Table S4: Working hours. Table S5: Attitudes to the use of homes among community nurses vs. hospital nurses. Table S6: Disagreeing with the use of patients’ home, regression using individual items. Table S7: Disagreeing with the use of institution, regression using individual items. Table S8: SEM model of home nurses disagreeing with the use of patients’ home.
