# Supplementary material for: After Hospital: Should Older Care-Needing Patients Be Transferred to Their Homes or to an Intermediate Care Institution?
Source: Healthcare (Basel). 2022 Mar 3;10(3):475. doi: 10.3390/healthcare10030475 (PMC8955582; doi:10.3390/healthcare10030475)
Supplement: Supplementary file 1 [file healthcare-10-00475-s001.zip › healthcare-1569482-supplementary.pdf]

Supplementary Materials for “After Hospital: Should Older Care-Needing Patients be Transferred to their Homes or to an Intermediate Care Institution?” by Gautun, Kvæl, Bratt

The Supplementary Materials are developed by Christopher Bratt

Contents

1 Descriptive Statistics **3**

2 Simple Probit Regression **5**

2.1 Age did not Explain Different Attitudes across Services 5

2.2 Results for a Model with Individual Items 5

3 Structural Equation Modelling (Adding Latent Variables) **7**

3.1 Inadequate Staffing and Medical Equipment Predicting Opposition to the Use of Patients' Homes 7

3.2 Testing for a Method Effect 10

4 Code for Analyses **12**

List of Tables

S1 Age of participants . . . 3

S2 Years experience as a nurse . . . 3

S3 Years at the current work place . . . 4

S4 Working hours . . . 4

S5 Attitudes to the use of homes among community nurses vs hospital nurses . . . 5

S6 Disagreeing with the use of patients' home, regression using individual items . . . 6

S7 Disagreeing with the use of institution, regression using individual items . . . 6

S8 SEM model of home nurses disagreeing with the use of patients' home . . . 8

List of Figures

S1 SEM model of home nurses disagreeing with the use of patients' home, standardised estimates 7

S2 SEM model with revised factor model, standardised estimates . . . 9

S3 SEM model of nurses in nursing homes disagreeing with the use of institution . . . 10

S4 SEM model with revised factor model, unstandardised estimates . . . 11

S5 SEM model with added method factor (tendency to disagree), unstandardised estimates . . . 11

1 Descriptive Statistics

Below are descriptive statistics with background variables for each of the three groups: home nurses (n = 2,112), nurses in nursing homes (n = 2,169), and hospital nurses (n = 2,328). Nearly all nurses were female (94%, 96%, and 93% in the three respective groups).

Nurses in home nursing and nursing homes were overall of similar age (Table S1); they had similar length of experience as nurse (Table S2), and at the current workplace (Table S3. Nurses in home nursing tended to have somewhat more working hours than nurses in nursing homes (Table S4. Nurses in Hospitals were overall younger (Table S1) and had fewer years of experience (Table S2).

Table S1: Age of participants

	Home nurses		Nursing homes		Hospitals	
Label	Freq.	Percent	Freq.	Percent	Freq.	Percent
25 years or less	111	5.26	107	4.93	277	11.90
26-30 years	297	14.06	260	11.99	658	28.26
31-35 years	312	14.77	282	13.00	351	15.08
36-40 years	277	13.12	280	12.91	249	10.70
41-45 years	280	13.26	290	13.37	205	8.81
46-50 years	255	12.07	236	10.88	185	7.95
51-55 years	242	11.46	249	11.48	190	8.16
56-60 years	159	7.53	215	9.91	121	5.20
61 years or more	83	3.93	125	5.76	91	3.91
Missing values	96	4.55	125	5.76	1	0.04

Table S2: Years experience as a nurse

	Home nurses		Nursing homes		Hospitals	
Label	Freq.	Percent	Freq.	Percent	Freq.	Percent
0-2 years	200	9.47	190	8.76	400	17.18
3-5 years	294	13.92	247	11.39	564	24.23
6-10 years	393	18.61	365	16.83	465	19.97
11-15 years	370	17.52	373	17.20	300	12.89
16-20 years	312	14.77	344	15.86	219	9.41
More than 20 years	440	20.83	530	24.44	376	16.15
Missing values	103	4.88	120	5.53	4	0.17

Table S3: Years at the current work place

	Home nurses		Nursing homes	
Label	Freq.	Percent	Freq.	Percent
0-2 years	388	18.37	459	21.16
3-5 years	496	23.48	518	23.88
6-10 years	546	25.85	473	21.81
11-15 years	263	12.45	258	11.89
16-20 years	196	9.28	195	8.99
More than 20 years	124	5.87	144	6.64
Missing values	99	4.69	122	5.62

Table S4: Working hours

	Home nurses		Nursing homes	
Label	Freq.	Percent	Freq.	Percent
0-20 hours	45	2.13	58	2.67
21-30 hours	286	13.54	317	14.62
31-35 hours	569	26.94	664	30.61
36-38 hours	873	41.34	785	36.19
39-40 hours	163	7.72	163	7.51
More than 40 hours	68	3.22	48	2.21
Missing values	108	5.11	134	6.18

2 Simple Probit Regression

2.1 Age did not Explain Different Attitudes across Services

Table S5 shows how opposition to the use of home nursing differed across services (the table uses hospital nurses as reference group, resulting in negative estimates for home nurses and nurses in nursing homes). Nurses were overall younger in hospitals, but adding age as a covariate had little effect on the estimated effect by working in home nursing or nursing homes.

Table S5: Attitudes to the use of homes among community nurses vs hospital nurses

	Services compared, without age		Services compared, age added as predictor	
Variables	Est	95 prct CI	Est	95 prct CI
**Regression weights**				
Home nurses	-0.33	[-0.39, -0.26]	-0.30	[-0.36, -0.23]
Nursing homes	-0.62	[-0.68, -0.55]	-0.58	[-0.64, -0.51]
Age			-0.05	[-0.06, -0.03]
**Thresholds for the depenent**				
Threshold 1	-2.14	[-2.21, -2.07]	-2.32	[-2.40, -2.23]
Threshold 2	-1.33	[-1.38, -1.28]	-1.51	[-1.58, -1.44]
Threshold 3	0.32	[0.27, 0.36]	0.14	[0.07, 0.20]
Threshold 4	1.06	[1.00, 1.11]	0.89	[0.82, 0.95]

2.2 Results for a Model with Individual Items

Tables S6 and S7 show results from analyses with item-level predictors of community nurses' disagreement with the use of patients' homes (or the use of an institution). Home nurses and nurses in nursing homes were analysed separately. Variables such as age, years of experience, and working hours did not predict attitudes to the use of patients' homes or institution (see "Model 2" in Tables S6 and S7).

Tables S6 and S7 show that leaders were less inclined to disagree with the use of their own services, and leaders both in home nursing and in nursing homes were substantially less likely than non-leaders to disagree with the use of patients' homes. Further education among nurses (assessed with a four-point scale) was only a very weak predictor.

The remaining predictors in Table 1 refer to staffing problems and inadequate medical equipment. The analysis showed that the four indicators of staffing problems should not be estimated as separate predictors, as evident by the very low estimated effects for these items when used as separate predictors. Due to the close resemblance of these items, a more reasonable approach was to consider them as indicators of a latent variable: overall inadequate staffing of the service.

Table S6: Disagreeing with the use of patients' home, regression using individual items

	Home nurses				Nursing homes			
	Model 1		Model 2		Model 1		Model 2	
Predictors	Est	95 prct CI	Est	95 prct CI	Est	95 prct CI	Est	95 prct CI
Leader	-0.32	[-0.46, -0.19]	-0.30	[-0.44, -0.15]	-0.26	[-0.40, -0.12]	-0.23	[-0.38, -0.08]
Further education	-0.06	[-0.13, -0.00]	-0.04	[-0.11, 0.03]	-0.05	[-0.10, 0.01]	-0.03	[-0.09, 0.03]
Too few nurses	0.08	[0.03, 0.12]	0.08	[0.03, 0.12]	0.06	[0.02, 0.10]	0.06	[0.02, 0.10]
Too few other trained	0.02	[-0.02, 0.07]	0.02	[-0.03, 0.07]	0.02	[-0.03, 0.06]	0.02	[-0.03, 0.07]
Too many non-trained	0.05	[0.00, 0.09]	0.04	[-0.00, 0.09]	0.12	[0.07, 0.16]	0.12	[0.07, 0.16]
Too many vacancies	0.07	[0.03, 0.11]	0.07	[0.02, 0.11]	0.01	[-0.03, 0.05]	0.01	[-0.03, 0.05]
Inadequate medical equipment	0.16	[0.12, 0.21]	0.16	[0.12, 0.20]	0.07	[0.03, 0.12]	0.07	[0.03, 0.12]
Age			-0.01	[-0.04, 0.03]			-0.02	[-0.06, 0.01]
Years as nurse			-0.04	[-0.09, 0.02]			0.00	[-0.05, 0.06]
Years at current work place			-0.01	[-0.06, 0.03]			0.01	[-0.03, 0.06]
Working hours			0.01	[-0.05, 0.06]			-0.04	[-0.10, 0.01]

Table S7: Disagreeing with the use of institution, regression using individual items

	Home nurses				Nursing homes			
	Model 1		Model 2		Model 1		Model 2	
Predictors	Est	95 prct CI	Est	95 prct CI	Est	95 prct CI	Est	95 prct CI
Leader	0.07	[-0.07, 0.22]	0.04	[-0.11, 0.19]	-0.17	[-0.30, -0.03]	-0.16	[-0.31, -0.01]
Further education	0.08	[0.02, 0.13]	0.07	[0.00, 0.13]	-0.04	[-0.10, 0.01]	-0.03	[-0.09, 0.03]
Too few nurses	0.03	[-0.02, 0.07]	0.03	[-0.01, 0.07]	-0.02	[-0.07, 0.02]	-0.02	[-0.06, 0.02]
Too few other trained	-0.02	[-0.07, 0.02]	-0.03	[-0.08, 0.02]	-0.06	[-0.10, -0.01]	-0.05	[-0.09, -0.00]
Too many non-trained	0.02	[-0.02, 0.06]	0.02	[-0.03, 0.06]	0.03	[-0.01, 0.08]	0.03	[-0.02, 0.08]
Too many vacancies	-0.02	[-0.06, 0.02]	-0.01	[-0.05, 0.03]	0.03	[-0.01, 0.07]	0.02	[-0.02, 0.06]
Inadequate medical equipment	0.00	[-0.05, 0.04]	0.00	[-0.05, 0.04]	0.05	[0.00, 0.09]	0.04	[-0.00, 0.08]
Age			0.00	[-0.03, 0.04]			-0.02	[-0.05, 0.02]
Years as nurse			0.00	[-0.05, 0.05]			0.00	[-0.05, 0.05]
Years at current work place			0.02	[-0.03, 0.06]			0.00	[-0.04, 0.04]
Working hours			0.03	[-0.03, 0.08]			0.00	[-0.05, 0.05]

3 Structural Equation Modelling (Adding Latent Variables)

3.1 Inadequate Staffing and Medical Equipment Predicting Opposition to the Use of Patients' Homes

We were particularly interested in home nurses' opposition to the use of older patients' homes after hospital discharge. Figure S1 focuses on this group of nurses and shows the results of a model estimating inadequate staffing as a latent variable (called "staff" in Figure S1), adding an item on inadequate medical equipment as a separate predictor as well as an item distinguishing between nurses with or without a leader position. Table S8 shows detailed information on parameters in the model illustrated by Figure S1.

An alternative model of home nurses' opposition to the use of patients' homes was also tested: using medical equipment as a further indicator of the latent variable, making the latent variable a measure of overall inadequate resources (Figure S2).

A separate analysis focused on *nurses in nursing homes*, using their opposition to the use of institution as the dependent variable (Figure S3, based on the previous model estimating home nurses opposition to the use of their service for older patients discharged from hospital). As expected, and shown in Figure S3, the model used for home nurses' opposition to the use of their service was less applicable to views among nurses in nursing and opposition to the use of their service (Pseudo R^2 for the dependent variable = .13 among home nurses, but only .01 among nurses in nursing homes.

Leaders saw less problems in staffing, b = -0.26 [-0.38, -0.15], and in medical equipment, b = -0.18 [-0.32, -0.04], than non-leaders did.

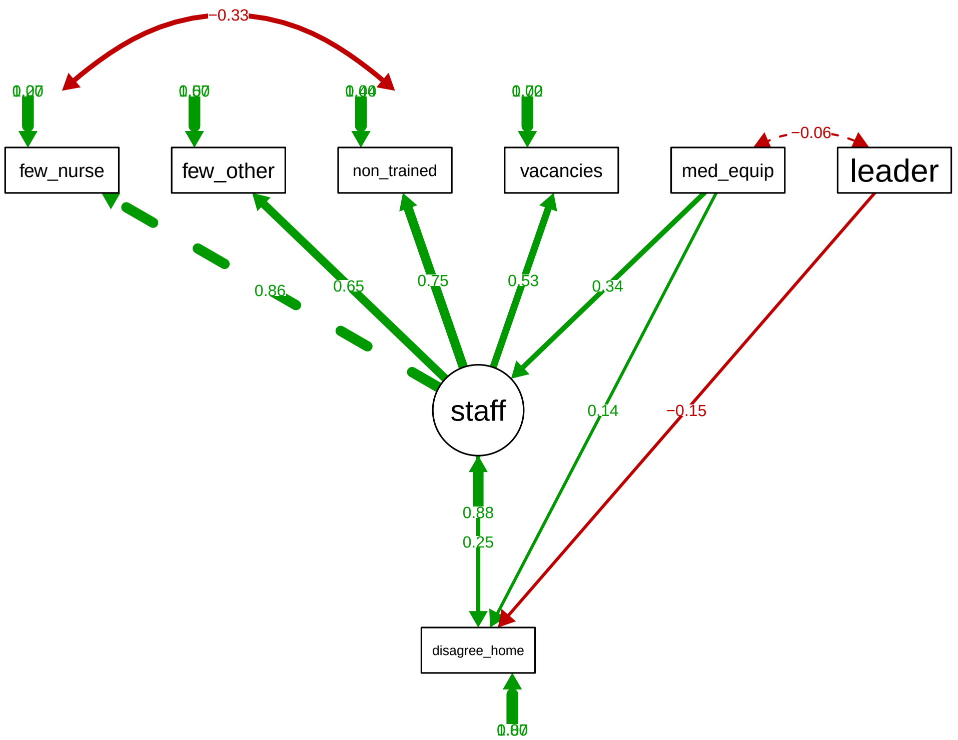

Figure S1: SEM model of home nurses disagreeing with the use of patients' home, standardised estimates

Table S8: SEM model of home nurses disagreeing with the use of patients' home

Predictors	Est	95 prct CI	Beta
**Factor loadings**			
Too few nurses	1.00	[1.00, 1.00]	0.86
Too few other trained	0.75	[0.68, 0.82]	0.65
Too many non-trained	0.87	[0.81, 0.92]	0.75
Too many vacancies	0.60	[0.54, 0.67]	0.53
**Correlated residuals**			
Too few nurses, Too many untrained	-0.12	[-0.18, -0.06]	-0.33
**Regression weights: Disagreeing with use of patients' home**			
Leader	-0.43	[-0.56, -0.30]	-0.15
Inadequate staffing	0.29	[0.23, 0.35]	0.25
Inadequate medical equipment	0.13	[0.09, 0.17]	0.14
**Regression weights: Inadequate staffing**			
Inadequate medical equipment	0.26	[0.23, 0.30]	0.34
**Thresholds for the depenent**			
Threshold 1	-1.28	[-1.43, -1.12]	-1.23
Threshold 2	-0.52	[-0.65, -0.38]	-0.49
Threshold 3	1.24	[1.10, 1.38]	1.19
Threshold 4	2.05	[1.89, 2.20]	1.97
**Residual**			
Disagreeing with use of home	0.94	[0.94, 0.94]	0.87
**Covariance**			
Leader, Inadequate medical equipment	-0.02	[-0.02, -0.02]	-0.06
**Model fit**			
Chi-square	63.65		
df	11.00		
p-value	0.00		
Comparative Fit Index (CFI)	0.98		
Root Mean Square Error of Approximation (RMSEA)	0.05		
RMSEA, lower CI limit	0.04		
RMSEA, upper CI limit	0.06		
Standardised Root Mean Squared Residual	0.02		

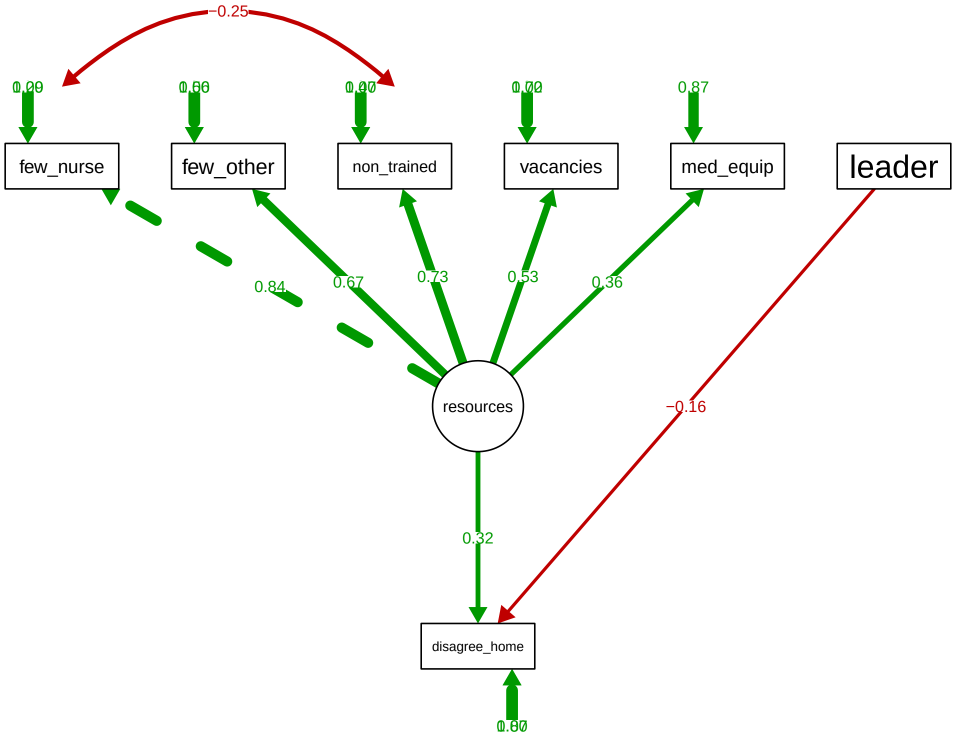

Figure S2: SEM model with revised factor model, standardised estimates

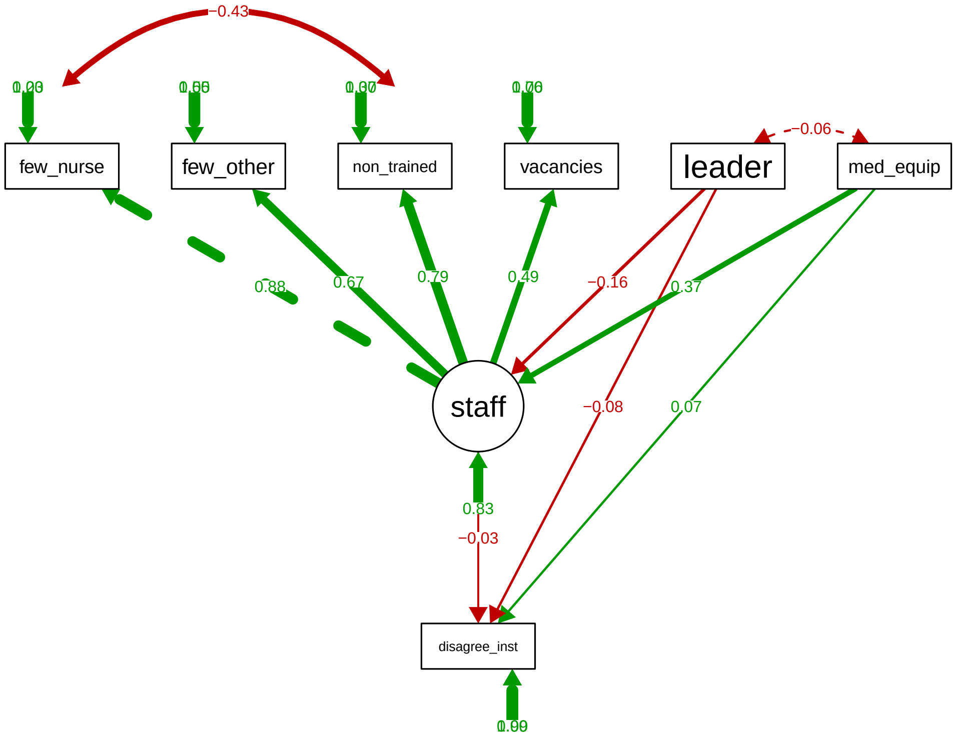

Figure S3: SEM model of nurses in nursing homes disagreeing with the use of institution

3.2 Testing for a Method Effect

Theoretically, the association between opposition to the use of patients homes and reported inadequate staffing (or inadequate medical equipment) could be an artefact of a method effect, that is, be biased by some nurses having a tendency to express negative views. We tested for this possibility by introducing a factor representing such a method effect in the analysis.

Figures S4 and S5 help test for such a method effect. Figure S4 repeats the model in Figure S2, but uses unstandardised estimates. These unstandardised estimates can be compared with the estimates in a model that tested for a possible method effect of a tendency to express disagreement or opposition (Figure S5). A comparison of Figures S4 and S5 indicated that the estimated association between reporting inadequate staffing and opposition to the use of patients' home was *not* an artefact of a method effect. The estimated association between the latent variable "resources" and the dependent variable actually increased when the model tested for a method effect.

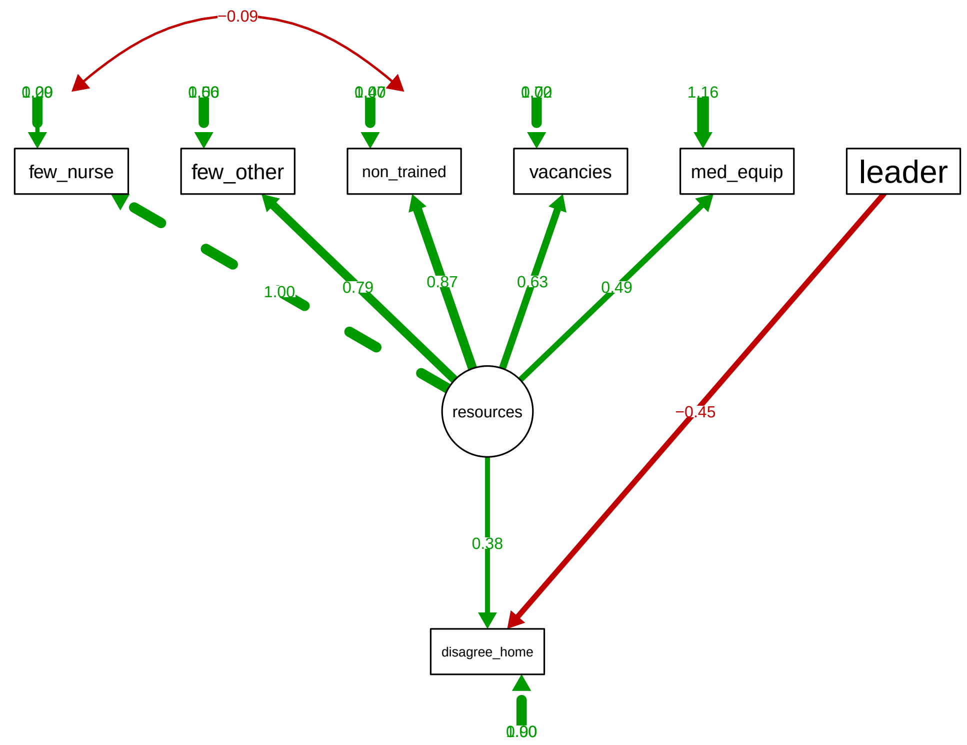

Figure S4: SEM model with revised factor model, unstandardised estimates

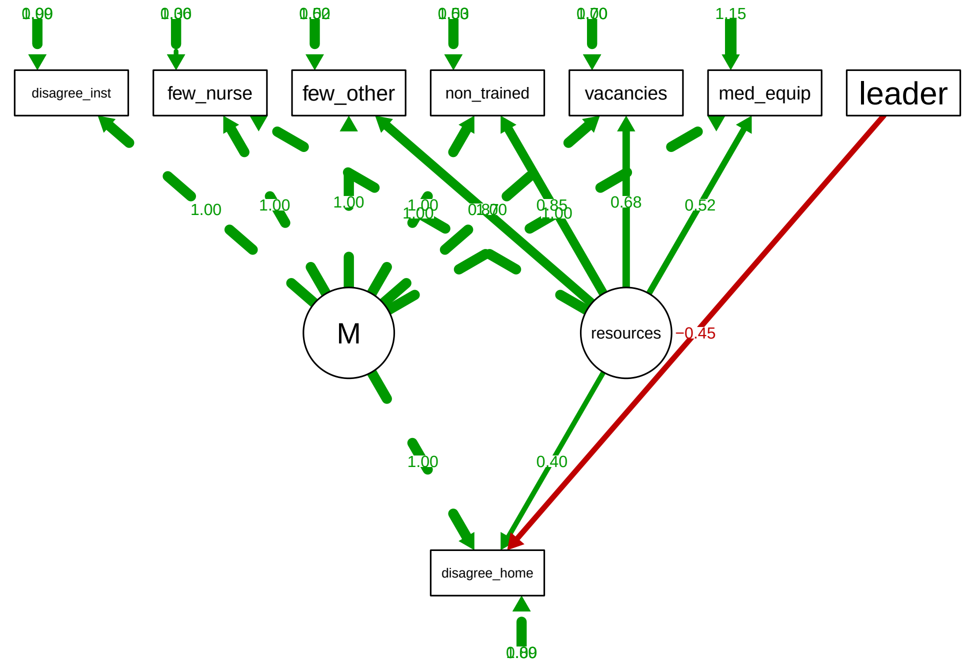

Figure S5: SEM model with added method factor (tendency to disagree), unstandardised estimates

4 Code for Analyses

```
# ---------------------------------------------------------------------------------- #
#                       Define defaults for knitr and bookdown                        #
# ---------------------------------------------------------------------------------- #

library(knitr)
library(bookdown)
# Set global knit options
opts_chunk$set(
  cache=FALSE, echo=FALSE,
  message=FALSE, warning=FALSE,
  error=TRUE, results=FALSE, warn.conflicts=FALSE,
  fig.align = 'center', quietly=TRUE,
  dpi=300
)

knit_hooks$set(inline = function(x) {
  prettyNum(x, big.mark = ",")
})

opts <- options(knitr.kable.NA = "") # "NA" in a table is printed as an empty cell
options(scipen=999)


# ---------------------------------------------------------------------------------- #
#                                   Load R packages                                   #
# ---------------------------------------------------------------------------------- #

# Load packages
pacman::p_load(plyr
             , tidyverse
             , sjlabelled
             , kableExtra
             , sjmisc
             , lavaan
             , semPlot
             , here # Point to the current working directory
)

# Solve potential conflicts between packages
library(conflicted)
conflict_prefer("here",       "here")
conflict_prefer("select",     "dplyr")
conflict_prefer("rename",     "dplyr")
conflict_prefer("arrange",    "dplyr")
conflict_prefer("filter",     "dplyr")
conflict_prefer("summarise",  "dplyr")
conflict_prefer("mutate",     "dplyr")
conflict_prefer("group_rows", "kableExtra")


# ---------------------------------------------------------------------------------- #
```

```r
#                              Define custom functions                                  #
# ------------------------------------------------------------------------------------ #

# -Function-
# Develop a frequency table
frq_table <- function(x, title = " ") {
  data <- Home_Nurses |> select(paste(x))
  tab1 <- data.frame(frq(data))

  data <- Nursing_Homes |> select(paste(x))
  tab2 <- data.frame(frq(data))

  data <- Hospitals |> select(paste(x))
  tab3 <- data.frame(frq(data))

  tab <- cbind(tab1[, 3:5], tab2[, 4:5], tab3[, 4:5])
  tab <- repair_names(tab)

  # Remove columns for Hospitals if no data available for these nurses
  if (x %in% c("years_current", "work_hours")) {
    tab <- tab[, 1:5]
  }
  # Add label "Missing values"
  line_nr <- nrow(tab)
  tab[line_nr, 1] <- "Missing values"

  # Write table with kableExtra
  if (x %in% c("years_current", "work_hours")) {
    kbl(tab, booktabs = T, linesep = "", digits = 2,
        caption = title,
        col.names = c("Label", "Freq.", "Percent",
                      "Freq.", "Percent")) |>
        add_header_above(c(" ", "Home nurses" = 2,
                           "Nursing homes" = 2)) |>
        kable_styling(font_size = 9, latex_options = "hold_position")
  } else {
    kbl(tab, booktabs = T, linesep = "", digits = 2,
        caption = title,
        col.names = c("Label", "Freq.", "Percent",
                      "Freq.", "Percent",
                      "Freq.", "Percent")) |>
        add_header_above(c(" ", "Home nurses" = 2,
                           "Nursing homes" = 2,
                           "Hospitals" = 2)) |>
        kable_styling(font_size = 9, latex_options = "hold_position")
  }
}

# -Function-
# Run lavaan, reading results, and preparing sections of a table
run_lavaan <- function(data, model) {
   out <- sem(data=data, model=model, ordered=ordered, missing="pairwise")
   para <- parameterEstimates(out, ci = T, standardized = T)
```

```
   para <- para |> filter(op == "~")
   para <- para |> select(rhs, est, ci.lower, ci.upper, std.nox)

   # Combine CI columns into [lower, upper]
   ci   <- para |> select(ci.lower, ci.upper)
   ci   <- round(ci, digits = 2)
   ci   <- within(ci, CI <- sprintf("[%.02f, %.02f]", ci.lower, ci.upper))
   ci   <- ci |> select(CI)
   para <- data.frame(para, ci)
   para <- para |> select(rhs, est, CI, std.nox)
}

# -Function-
# Use semPaths() from the semPlot package with customised arguments
semPaths2 <- function(input, what = "paths", layout = "tree") {
  semPaths(input
         , what = what
         , style = "lisrel"
         , layout = layout
         , nCharNodes = 0
         , sizeMan    = 10
         , sizeMan2   = 4
         , thresholds = F
         , intercepts = F
         , fade = F
         )
}


# ---------------------------------------------------------------------------------- #
#                                          Data                                      #
# ---------------------------------------------------------------------------------- #

Data_Nurses <- read_stata("After_Hospital.dta")

# Data frames used for descriptive statistics
Home_Nurses   <- Data_Nurses |> filter(workplace2 == 1)
Nursing_Homes <- Data_Nurses |> filter(workplace2 == 2)
Hospitals     <- Data_Nurses |> filter(workplace2 == 3)


# ---------------------------------------------------------------------------------- #
#                                 Descriptive Statistics                             #
# ---------------------------------------------------------------------------------- #

# Age of participants
frq_table("age", "Age of participants")
# Years experience as a nurse
frq_table("years_nurse", "Years experience as a nurse")
# Years at current workplace
frq_table("years_current", "Years at the current work place")
# Working hours
frq_table("work_hours", "Working hours")
```

```r
# ------------------------------------------------------------------------------- #
#          Simple probit and testing for the effect of age                         #
# ------------------------------------------------------------------------------- #

# Define ordered variables (only one of these variables is used in each model)
ordered <- c("disagree_home", "disagree_inst")

# Testing for group differences (hospital nurses as reference group)
mod_group_home <- c("disagree_home ~ home_nurses + nursing_homes")
mod_group_inst <- c("disagree_inst ~ home_nurses + nursing_homes")

out_group_home <- sem(mod_group_home, data = Data_Nurses, ordered = ordered)
out_group_inst <- sem(mod_group_inst, data = Data_Nurses, ordered = ordered)
summary(out_group_home, ci=TRUE)
summary(out_group_inst, ci=TRUE)

# Test effect of age as a predictor (disagreeing with using patients' homes)
mod_compare_services1 <- c("disagree_home ~ home_nurses + nursing_homes")
mod_compare_services2 <- c("disagree_home ~ home_nurses + nursing_homes + age")

out_compare_services1 <- sem(mod_compare_services1, data=Data_Nurses,
                             ordered = ordered)
out_compare_services2 <- sem(mod_compare_services2, data=Data_Nurses,
                             ordered = ordered)

para_compare1 <- parameterEstimates(out_compare_services1)[1:6, ]
para_compare2 <- parameterEstimates(out_compare_services2)[1:7, ]

# Combine CI columns into [lower, upper]
ci    <- para_compare1 |> select(ci.lower, ci.upper)
ci    <- round(ci, digits = 2)
ci    <- within(ci, CI <- sprintf("[%.02f, %.02f]", ci.lower, ci.upper))
ci    <- ci |> select(CI)
para_compare1 <- data.frame(para_compare1, ci)
para_compare1 <- para_compare1 |> select(-se, -z,
                                         -pvalue, -ci.lower, -ci.upper)

ci    <- para_compare2 |> select(ci.lower, ci.upper)
ci    <- round(ci, digits = 2)
ci    <- within(ci, CI <- sprintf("[%.02f, %.02f]", ci.lower, ci.upper))
ci    <- ci |> select(CI)
para_compare2 <- data.frame(para_compare2, ci)
para_compare2 <- para_compare2 |> select(-se, -z,
                                         -pvalue, -ci.lower, -ci.upper)

# Add an empty row for variable added in Model 2
para_compare1 <- para_compare1 |>
  add_row(.after = 2)

para_compare <- cbind(para_compare2[, 1:3], para_compare1[, c(4:5)]
                      , para_compare2[, c(4:5)])
```

```r
variables_compare <- c("Home nurses"
                     , "Nursing homes"
                     , "Age"
                     , "Threshold 1"
                     , "Threshold 2"
                     , "Threshold 3"
                     , "Threshold 4")

para_compare <- cbind(variables_compare, para_compare[, 4:7])

kable(para_compare, booktabs = T, linesep = "", digits = 2,
  caption = "Attitudes to the use of homes among community nurses vs hospital nurses",
  col.names = c("Variables", "Est", "95 prct CI", "Est", "95 prct CI")) |>
  add_header_above(c(" ", "Services compared, without age" = 2,
                        "Services compared, age added as predictor" = 2)) |>
  group_rows("Regresion weights", 1, 3) |>
  group_rows("Thresholds for the depenent", 4, 7) |>
  kable_styling(font_size = 9, latex_options = "hold_position")

# The table above did not directly compare home nurses and
# nurses in home nursing. These two groups are compared below.
Comm_Nurses <- Data_Nurses |>
  filter(workplace2 %in% c(1, 2)) |>
  mutate(w_place = workplace2 - 1)

comm1_out <- sem(data = Comm_Nurses, model = "disagree_home ~ w_place",
                ordered="disagree_home")
comm2_out <- sem(data = Comm_Nurses, model = "disagree_inst ~ w_place",
                ordered="disagree_inst")

summary(comm1_out)
summary(comm2_out)


# ---------------------------------------------------------------------------------- #
#                          Full model, using individual items                          #
# ---------------------------------------------------------------------------------- #

# Analyses below focus on one group of nurses, either home nurses
# or nurses in nursing homes.

# Define workplace-specific data frames and models to test
Sample_Hn <- Data_Nurses |> filter(workplace2 == 1)
Sample_Nh <- Data_Nurses |> filter(workplace2 == 2)

# Models testing attitudes to sending patients to their homes
mod_home_1 <- c("disagree_home ~ leader + furth_edu + few_nurse + few_other +
                              non_trained + vacancies + med_equip")
mod_home_2 <- c(mod_home_1,
                "disagree_home ~ age + years_nurse + years_current + work_hours")

# Models testing attitudes to sending patients to a nursing home
mod_inst_1 <- c("disagree_inst ~ leader + furth_edu + few_nurse + few_other +
```

```
                                non_trained + vacancies + med_equip")
mod_inst_2 <- c(mod_inst_1,
                "disagree_inst ~ age + years_nurse + years_current + work_hours")

# Disagreeing with the use of patients' home (drop standardized estimates from results)
res_Hn_home_1 <- run_lavaan(Sample_Hn, mod_home_1) |> select(-std.nox)
res_Hn_home_2 <- run_lavaan(Sample_Hn, mod_home_2) |> select(-std.nox)
res_Nh_home_1 <- run_lavaan(Sample_Nh, mod_home_1) |> select(-std.nox)
res_Nh_home_2 <- run_lavaan(Sample_Nh, mod_home_2) |> select(-std.nox)

# Disagreeing with the use of an institution (drop standardized estimates from results)
res_Hn_inst_1 <- run_lavaan(Sample_Hn, mod_inst_1) |> select(-std.nox)
res_Hn_inst_2 <- run_lavaan(Sample_Hn, mod_inst_2) |> select(-std.nox)
res_Nh_inst_1 <- run_lavaan(Sample_Nh, mod_inst_1) |> select(-std.nox)
res_Nh_inst_2 <- run_lavaan(Sample_Nh, mod_inst_2) |> select(-std.nox)

# Prepare table of disagreement with the use of patients' home
res_home <-
  res_Hn_home_1 |>
  full_join(res_Hn_home_2, by = "rhs") |>
  full_join(res_Nh_home_1, by = "rhs") |>
  full_join(res_Nh_home_2, by = "rhs")
kbl(res_home, "rst", digits = 2)

res_inst <-
  res_Hn_inst_1 |>
  full_join(res_Hn_inst_2, by = "rhs") |>
  full_join(res_Nh_inst_1, by = "rhs") |>
  full_join(res_Nh_inst_2, by = "rhs")
kbl(res_inst, "rst", digits = 2)

variables_res <- c("Leader"
                 , "Further education"
                 , "Too few nurses"
                 , "Too few other trained"
                 , "Too many non-trained"
                 , "Too many vacancies"
                 , "Inadequate medical equipment"
                 # Additional predictors, verified to be of little relevance
                 , "Age"
                 , "Years as nurse"
                 , "Years at current work place"
                 , "Working hours")

res_home <- cbind(variables_res, res_home[, -1])
res_inst <- cbind(variables_res, res_inst[, -1])

# Print tables
kbl(res_home, booktabs = T, linesep = "", digits = 2,
  caption = "Disagreeing with the use of patients' home, regression using individual items",
  col.names = c("Predictors", "Est", "95 prct CI"
                            , "Est", "95 prct CI"
                            , "Est", "95 prct CI"
```

```r
                           , "Est", "95 prct CI")) |>
  add_header_above(c(" ", "Model 1" = 2, "Model 2" = 2
                         , "Model 1" = 2, "Model 2" = 2)) |>
  add_header_above(c(" ", "Home nurses" = 4, "Nursing homes" = 4)) |>
  kable_styling(font_size = 8, latex_options = "hold_position")

kbl(res_inst, booktabs = T, linesep = "", digits = 2,
  caption = "Disagreeing with the use of institution, regression using individual items",
  col.names = c("Predictors", "Est", "95 prct CI"
                           , "Est", "95 prct CI"
                           , "Est", "95 prct CI"
                           , "Est", "95 prct CI")) |>
  add_header_above(c(" ", "Model 1" = 2, "Model 2" = 2
                         , "Model 1" = 2, "Model 2" = 2)) |>
  add_header_above(c(" ", "Home nurses" = 4, "Nursing homes" = 4)) |>
  kable_styling(font_size = 8, latex_options = "hold_position")







# -------------------------------------------------------------------------------- #
#                      Structural Equation Modelling                               #
# -------------------------------------------------------------------------------- #

# Focusing on nurses i home nursing, first estimating a factor model with good fit.
# Define which items are ordered in subsequent SEM analyses and fit indices
ordered <- c("disagree_home", "disagree_inst", "few_nurse", "few_other",
             "non_trained", "vacancies")

fit_indices <- c("chisq.scaled", "df", "pvalue.scaled", "cfi.scaled", "rmsea.scaled",
                 "rmsea.ci.lower.scaled", "rmsea.ci.upper.scaled", "srmr")


# - Measurement model of staffing problems -
# CFA model of staffing problems
mod_cfa <- c("staff =~ few_nurse + few_other + non_trained + vacancies")
res_cfa <- cfa(mod_cfa, data = Sample_Hn, ordered = ordered,
               missing = "pairwise")
fitMeasures(res_cfa, fit_indices)
residuals(res_cfa)
modindices(res_cfa) |> arrange(-mi)

# CFA with added residual covariance increasing fit
mod_cfa <- c(mod_cfa, "few_nurse ~~ non_trained")
res_cfa <- cfa(mod_cfa, data = Sample_Hn, ordered = ordered,
               missing = "pairwise")
fitMeasures(res_cfa, fit_indices)

# - Differences between leaders and non-leaders -
# Test of differences in views on staffing dependent on leader position
# Testing difference in views on in the latent variable "staff" and medical equipment
```

```r
mod_diff_leader <- c(mod_cfa, "staff ~ leader; med_equip ~ leader; staff ~~med_equip")
res_diff_leader <- cfa(mod_diff_leader, data = Sample_Hn, ordered = ordered,
                     missing = "pairwise")
fitMeasures(res_diff_leader, fit_indices)
para_diff_leader <- parameterEstimates(res_diff_leader)
para_diff_leader <- para_diff_leader |> filter(rhs == "leader", op == "~")
para_diff_leader
# Combine CI columns into [lower, upper]
ci   <- para_diff_leader |> select(ci.lower, ci.upper)
ci   <- round(ci, digits = 2)
ci   <- within(ci, CI <- sprintf("[%.02f, %.02f]", ci.lower, ci.upper))
ci   <- ci |> select(CI)
para_diff_leader <- data.frame(para_diff_leader, ci)
para_diff_leader <- para_diff_leader |> select(lhs, op, rhs, est, CI)
# Resulting parameters
para_diff_leader[1, 4]; para_diff_leader[1, 5] # Staffing
para_diff_leader[2, 4]; para_diff_leader[2, 5] # Medical equipment

# - Full SEM model -
# SEM model: First attempt
mod_sem <- c("staff =~ few_nurse + few_other + non_trained + vacancies
              few_nurse ~~ non_trained
              disagree_home ~ leader + staff + med_equip
             ")
res_sem <- sem(mod_sem, data = Sample_Hn, ordered = ordered,
                     missing = "pairwise")
fitMeasures(res_sem, fit_indices)
residuals(res_sem)$res.cov
modindices(res_sem) |> arrange(-mi)

# The SEM model needed minor modification to achieve fit. We identified two
# models that gave sufficient fit.

# - Solution 1 -
# Add a causal path from inadequate medical equipment to inadequate
# staffing (as suggested by modification indices).
# Model definition includes estimations of indirect and total effects.

mod_sem1 <- c("
               staff     =~ few_nurse + few_other + non_trained + vacancies
               few_nurse     ~~ non_trained
               disagree_home ~  leader

               disagree_home ~ b*staff
               disagree_home ~ c*med_equip
               staff         ~ a*med_equip

               # indirect effect (a*b)
                 ab := a*b
               # total effect
                 total := c + (a*b)
             ")
out_sem1 <- sem(mod_sem1, data = Sample_Hn, ordered = ordered,
```

```
                missing = "pairwise")
fitMeasures(out_sem1, fit_indices)
parameterEstimates(out_sem1)
inspect(out_sem1, "r2") # R2 = .132

# - Solution 2 -
# Redefine the factor for inadequate staffing to inadequate resources
# The model gives sufficient fit, but does not distinguish between
# staffing problems and medical equipment.
mod_sem2 <- c("resources =~ few_nurse + few_other + non_trained + vacancies
                           + med_equip
              few_nurse  ~~ non_trained
              disagree_home ~ leader + resources")

res_sem2 <- sem(mod_sem2, data = Sample_Hn, ordered = ordered,
                     missing = "pairwise")

fitMeasures(res_sem2, fit_indices)
inspect(res_sem2, "r2") # R2 = .130

# - Solution 1 -
# Solution 1 is used to collect parameters
para_sem <- parameterEstimates(out_sem1, standardized = T)

para_sem <- para_sem |>
  filter( op %in% c("=~", "~") | lhs=="disagree_home" | (op=="~~" & lhs!=rhs)) |>
  filter(!op %in% c("~*~", "~1")) |>
  select(lhs, op, rhs, est, ci.lower, ci.upper, std.all)

# Combine CI columns into [lower, upper]
ci    <- para_sem |> select(ci.lower, ci.upper)
ci    <- round(ci, digits = 2)
ci    <- within(ci, CI <- sprintf("[%.02f, %.02f]", ci.lower, ci.upper))
ci    <- ci |> select(CI)
para_sem <- data.frame(para_sem, ci)
para_sem <- para_sem |> select(lhs, op, rhs, est, CI, std.all)

# Model fit
fit <- data.frame(fitMeasures(out_sem1, fit_indices))
fit <- rownames_to_column(fit)
names(fit) <- c("rhs", "est")

para_sem <- rbind.fill(para_sem, fit)

variables_sem <- c("Too few nurses"
                 , "Too few other trained"
                 , "Too many non-trained"
                 , "Too many vacancies"
                 , "Too few nurses, Too many untrained"
                 , "Leader"
                 , "Inadequate staffing"
                 , "Inadequate medical equipment"
                 , "Inadequate medical equipment"
```

```
                  , "Threshold 1"
                  , "Threshold 2"
                  , "Threshold 3"
                  , "Threshold 4"
                  , "Disagreeing with use of home"
                  , "Leader, Inadequate medical equipment"
                  , "Chi-square"
                  , "df"
                  , "p-value"
                  , "Comparative Fit Index (CFI)"
                  , "Root Mean Square Error of Approximation (RMSEA)"
                  , "RMSEA, lower CI limit"
                  , "RMSEA, upper CI limit"
                  , "Standardised Root Mean Squared Residual")

para_sem <- cbind(variables_sem, para_sem[, 4:6])

# Print path diagram of the model used
semPaths2(out_sem1, "stand", "tree")

kbl(para_sem, booktabs = T, linesep = "", digits = 2,
  caption = "SEM model of home nurses disagreeing with the use of patients' home",
  col.names = c("Predictors", "Est", "95 prct CI", "Beta"))                       |>
  group_rows("Factor loadings", 1, 4)                                              |>
  group_rows("Correlated residuals", 5, 5)                                         |>
  group_rows("Regression weights: Disagreeing with use of patients' home", 6, 8)  |>
  group_rows("Regression weights: Inadequate staffing", 9, 9)                      |>
  group_rows("Thresholds for the depenent", 10, 13)                                |>
  group_rows("Residual", 14, 14)                                                   |>
  group_rows("Covariance", 15, 15)                                                 |>
  group_rows("Model fit", 16, 23)                                                  |>
  kable_styling(font_size = 8, latex_options = "HOLD_position")

# Print path diagram of the model with extended factor, standardised estimates
semPaths2(res_sem2, "stand", "tree")

mod_method <- ("
               M =~  1*disagree_inst  +
                     1*disagree_home  +
                     1*few_nurse      +
                     1*few_other      +
                     1*non_trained    +
                     1*vacancies      +
                     1*med_equip

               resources =~ few_nurse + few_other + non_trained + vacancies + med_equip

               M ~~ 0*resources
               disagree_home ~ leader + resources

               ")

out_method <- sem(mod_method, data = Sample_Hn, ordered = ordered, missing = "pairwise")
```

```
fitMeasures(out_method)
modindices(out_method) |> arrange(-mi)

# SEM model, Nursing homes
# This analysis focuses on nurses i nursing homes

mod_semNH <- c("
               staff        =~ few_nurse + few_other + non_trained + vacancies
               few_nurse     ~~ non_trained
               disagree_inst ~  leader

               staff         ~ a*med_equip
               disagree_inst ~ b*staff
               disagree_inst ~ c*med_equip

               staff         ~ leader

               # indirect effect (a*b)
                 ab := a*b
               # total effect
                 total := c + (a*b)
             ")
out_semNH <- sem(mod_semNH, data = Sample_Nh, ordered = ordered,
                missing = "pairwise")
fitMeasures(out_semNH, fit_indices)
inspect(out_semNH, "r2") # R2 = .01
parameterEstimates(out_semNH, standardized=T)

# Model fit
fit <- data.frame(fitMeasures(out_semNH, fit_indices))
fit <- rownames_to_column(fit)
names(fit) <- c("rhs", "est")
fit

summary(out_semNH, standardized=T)
fitMeasures(out_semNH, fit_indices)

# Print path diagram of nurses in nursing homes disagreeing
# with the use of institution
semPaths2(out_semNH, "stand")

# Print path diagram of the model with extended factor, unstandardised estimates
semPaths2(res_sem2, "est", "tree")

# Print path diagram of test of potential method effect
semPaths2(out_method, "est", "tree")
```
